# In Silico Identification of Potential Druggable Binding Sites on CIN85 SH3 Domain

**DOI:** 10.3390/ijms22020534

**Published:** 2021-01-07

**Authors:** Serena Vittorio, Thomas Seidel, Arthur Garon, Rosaria Gitto, Thierry Langer, Laura De Luca

**Affiliations:** 1 Department of Chemical, Biological, Pharmaceutical and Environmental Sciences, University of Messina, Viale Palatucci 13, I-98168 Messina, Italy; rgitto@unime.it; 2 Department of Pharmaceutical Chemistry, University of Vienna, Althanstrasse 14, A-1090 Vienna, Austria; thomas.seidel@univie.ac.at (T.S.); arthur.garon@univie.ac.at (A.G.); thierry.langer@univie.ac.at (T.L.)

**Keywords:** protein-protein interactions, MUC1-CIN85, binding sites, molecular dynamics simulation, anticancer agents

## Abstract

Protein-protein interactions (PPIs) play a pivotal role in the regulation of many physiological processes. The dysfunction of some PPIs interactions led to the alteration of different biological pathways causing various diseases including cancer. In this context, the inhibition of PPIs represents an attractive strategy for the design of new antitumoral agents. In recent years, computational approaches were successfully used to study the interactions between proteins, providing useful hints for the design of small molecules able to modulate PPIs. Targeting PPIs presents several challenges mainly due to the large and flat binding surface that lack the typical binding pockets of traditional drug targets. Despite these hurdles, substantial progress has been made in the last decade resulting in the identification of PPI modulators where some of them even found clinical use. This study focuses on MUC1-CIN85 PPI which is involved in the migration and invasion of cancer cells. Particularly, we investigated the presence of druggable binding sites on the CIN85 surface which provided new insights for the structure-based design of novel MUC1-CIN85 PPI inhibitors as anti-metastatic agents.

## 1. Introduction

In the crowded cellular environment, many biological processes such as cellular growth, DNA replication, transcription, translation, and signal transduction are regulated by protein-protein interactions (PPIs) [1]. Different diseases, including cancer, are characterized by dysregulated PPIs that therefore represent valuable targets for the design of new therapeutic agents [2]. However, targeting PPIs by small molecules presents several challenges associated with the large and flat binding surface and the absence of well-defined cavities able to bind low molecular weight compounds with high affinity [3]. Furthermore, PPIs lack natural substrates or effectors that could serve as a starting point for the design of new ligands [4]. Despite these difficulties, several PPI inhibitors have been reported in the last decade and some of them were approved for clinical usage or are currently investigated in clinical trials [5]. A significant development related to the structural and energetic features of the binding surface was the observation that crucial residues named “hotspots” contribute to most of the binding energy [6]. Hotspots are usually located at the center of the interface and cover an area of which the dimension is comparable to that of a small molecule [7]. Targeting hotspots is one of the most frequently used strategies to disrupt PPIs and resulted in the successful identification of compounds with good binding affinity [7,8]. Moreover, due to the flexibility of the protein interface, the opening of transient pockets, that might provide suitable sites for small molecules, was observed [9,10,11].

**Figure 1:**
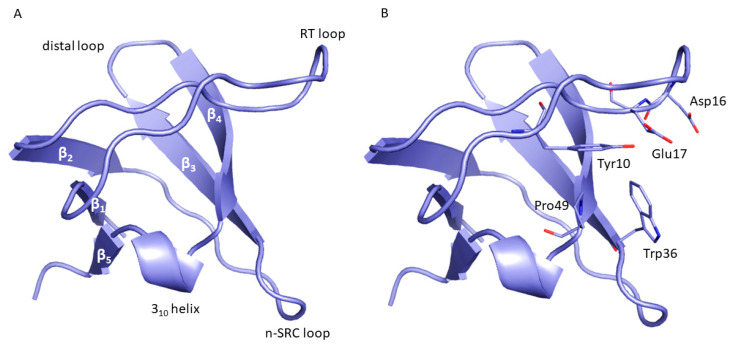
(**A**) Structure of Src homology 3 (SH3) domains. (**B**) The main residues involved in proline-rich motif recognition are displayed as sticks.

This study is focused on the MUC1-CIN85 PPI involved in the formation of several tumor metastasis [12]. It is well known that MUC1 is a transmembrane glycoprotein overexpressed in most adenocarcinomas. The extracellular domain of MUC1 is characterized by a variable number of tandem repeats (VNTRs), that are rich in prolines and highly glycosylated in physiological conditions. It has been demonstrated that VNTRs are hypo-glycosylated in cancer cells, thus increase the accessibility of the protein backbone to new PPIs that affect intracellular signaling in cancers when compared to normal cells [12,13]. CIN85 is a multifunctional adaptor protein that is involved in many biological processes such as signal transduction, cytoskeleton remodeling, endocytosis, and immunological synapse [14]. It is constituted by three Src homology 3 (SH3) domains, named as SH3A, SH3B, and SH3C, a proline-rich region, and a C-terminal coiled-coil domain. SH3 domains are small protein interaction modules, usually composed of approximately 60 amino acid residues, that bind to proline-rich sequences [15]. From a structural point of view, SH3 domains are composed of five β-strands, arranged in two antiparallel β-sheets, connected by three loops called RT, n-Src, and distal loops, and a short 3_10_ helix (Figure 1A). The canonical binding surface of SH3 domains is formed by the flat valley above β_3_ and β_4_ strands and the terminal portions of n-Src and RT loops [16,17]. The conserved amino acid residues that contribute to the recognition of proline-rich motifs are Tyr10, Trp36, and Pro49. Furthermore, an acidic pocket that might be involved in ionic interactions with the basic residues of the ligand is located in the RT loop and contains Asp16 and Glu17 residues (Figure 1B) [15,17].

The above-mentioned three domains of CIN85 are considered responsible for multiprotein complex formation. In particular, it has been established that the CIN85 SH3 domains recognize the atypical proline-arginine motif (PXXXPR) [18] in the ubiquitin ligase Cbl protein family (c-Cbl and Cbl-b), and in other CIN85 effectors [19]. X-ray structures confirmed that Cbl-b peptide accommodates two SH3 domains from separate CIN85 molecules and promotes the formation of Cbl-b/CIN85 ternary complex [17]. Moreover, mutation studies revealed the role of crucial additional residues in the proline-arginine motif of Cbl-b involved in the formation of this ternary complex [18].

In 2013, Cascio S. et al. reported for the first time the interaction between the tumor form of MUC1 and CIN85 [12] and showed that this association promotes the invasiveness of cancer cells and metastasis formation thus rendering it a viable target for pharmacological intervention in cancer therapy [12].

Based on the knowledge that the Cbl-b/CIN85 ternary complex is controlled by the atypical proline-arginine motif (PXXXPR), similarly, the proline-rich sequence PDTRPA of VNTRs of MUC1 proved to be a binding partner for SH3 domains of CIN85, thus paving the way to the identification of new ligands targeting CIN85 as MUC1 analogs [20].

Considering that an experimental structure of the complex is not available and assuming that compounds that bind to SH3 domains of CIN85 could prevent its association with MUC1, we performed an in silico study aimed at the identification of potential binding sites on the SH3 domain of CIN85. Starting from a 3D-protein structure, several algorithms have been developed that are able to detect putative ligand binding sites by employing a purely geometric approach or energy-based calculations [21]. These strategies depend on the atomic coordinates of the studied target and considering that proteins are dynamic systems, the static representation of a single set of coordinates could be too restrictive. Indeed, structural rearrangements could favor the exposure of druggable binding sites that may be missed when relying on just a single experimental structure [22]. Molecular dynamics (MD) simulations proved to be a useful technique to study the time evolution of biomolecular systems based on molecular mechanics, providing ensembles of protein conformations [23]. Herein, to account for protein flexibility, a molecular dynamics simulation was carried out to generate 32 representative conformers of the CIN85 SH3 N-terminal domain (SH3A). For each obtained structure, the presence of druggable binding sites were probed by employing two different strategies: (1) the geometry-based algorithms F-pocket [24] and (2) the computational mapping server FTMap [25]. As a result, we could identify a druggable binding site located on the CIN85 SH3 binding interface which was predicted in consensus by both applied methods. This pocket could be exploited for the structure- and fragment-based design of new ligands that might orthosterically inhibit MUC1-CIN85 PPI. Additional ligand binding sites that may serve as allosteric sites were also detected in other protein regions. Furthermore, this work focuses on the relevance of protein flexibility in binding site detection, thus highlighting the importance of MD simulations as a powerful tool to simulate protein motion thus enabling an incorporation of protein flexibility in structure-based drug design workflows.

## 2. Results and Discussion

**Figure 2:**
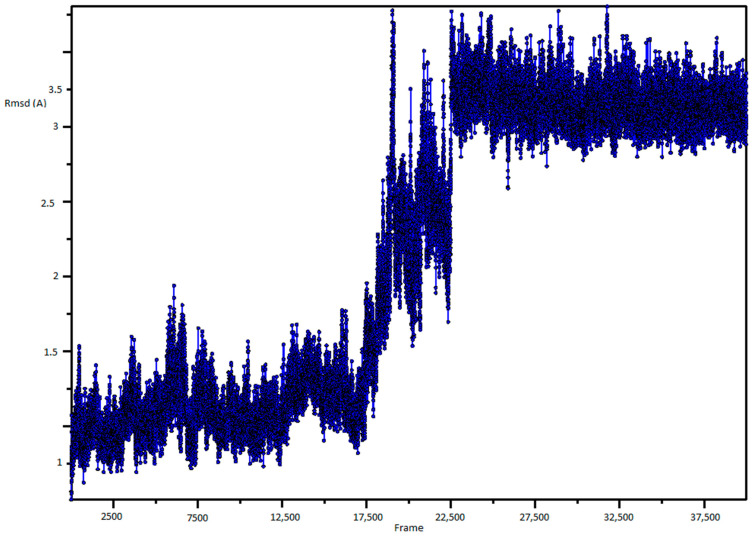
RMSD plot of protein backbone.

In the first step of our computational protocol, we performed a 400 ns MD simulation by using the software CHARMM [26] on the human CIN85 SH3 N-terminal domain, usually referred to as SH3A, extracted from the crystal structure of CIN85 in complex with the Cbl-b peptide (PDB code 2BZ8) [17]. The evolution of the root mean square deviations (RMSD) of the protein backbone was calculated and plotted as depicted in Figure 2. Fluctuations of the RMSD, mainly due to the motions of RT loop, occurred at 175 ns for about 30 ns after which the system remained stable for the rest of the simulation.

**Figure 3:**
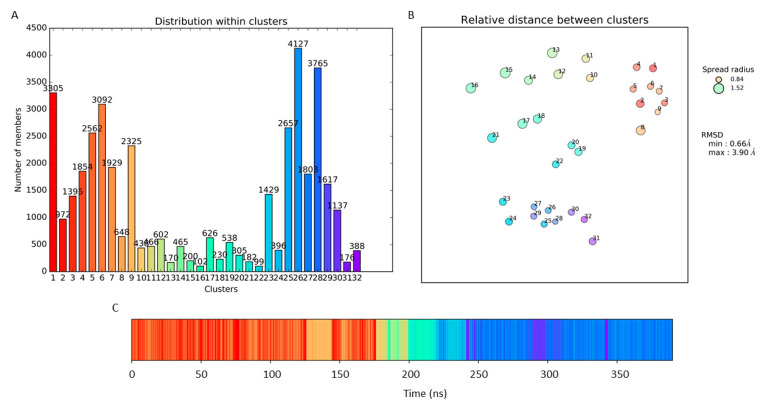
Results obtained from the clustering process. (**A**) Distribution of the frames within each cluster; (**B**) Relative distances between clusters based on the RMSD between representative frames; (**C**) Timeline barplot of the clustering. Each cluster is color coded.

The 40,000 frames obtained by the MD simulation were clustered according to RMSD using TTClust [27] which resulted in 32 clusters. A representative frame (the one with the lowest average RMSD in comparison to all other frames in the cluster) was identified for each cluster yielding 32 protein conformations. In Figure 3, the barplot related to the distribution of frames within each cluster (Panel A), the 2D projection plot of the relative distances between clusters (Panel B), and the timeline barplot showing the appearance of each frame during the simulation (Panel C), are displayed.

As shown in Figure 3A, cluster 26 (C26) is the most populated one, containing 4127 structures which appears almost at end of the simulation. The minimum and maximum distances between clusters (considering the RMSD between representative frames) are 0.66 Å and 3.90 Å, respectively, while the minimum and maximum spreads (average RMSD within clusters) are 0.84 and 1.52 (Figure 3B). The obtained structures were aligned with those of CIN85 SH3C domain solved by NMR experiments (PDB code 2K9G) [28] revealing a good superimposition between the structures as shown in Figure S1 of Supplementary Materials. We could observe small differences in the RT loop conformation that is twisted in some of the MD conformations when compared to the NMR models. 

The presence of binding sites suitable for accommodating a small molecule was probed both for the crystal structure and for each conformer obtained from the clustering procedure using F-Pocket2 [24] and the FTMap web server [25]. F-Pocket employs a geometry-based algorithm based on alpha sphere to detect ligand binding pockets; particularly, it calculates alpha spheres for the entire protein which are then clustered to identify any pockets. Small clusters with low quality are removed while nearby pockets are merged. Finally, the predicted pockets are scored and ranked based on their ability to accommodate small molecules [24]. Furthermore, F-pockets provides a druggability score between 0 and 1; a low score implies that drug-like molecules are not likely to bind to the pocket, while values higher than 0.5 (the threshold value) indicate that a binding could be possible [29]. FTMap is a computational mapping server for binding hotspots identification. It uses 16 small organic probes having different size and polarity to map protein surface. These probes are docked, clustered, and ranked according to their binding energies. The top-ranked clusters of different probes are grouped resulting in consensus sites (CSs) which represent potential binding sites. A binding site is considered druggable if the number of probe clusters within the CS is greater than 16 [25]. 

Concerning the crystal structure, no druggable binding sites were predicted by both methods.

**Figure 4:**
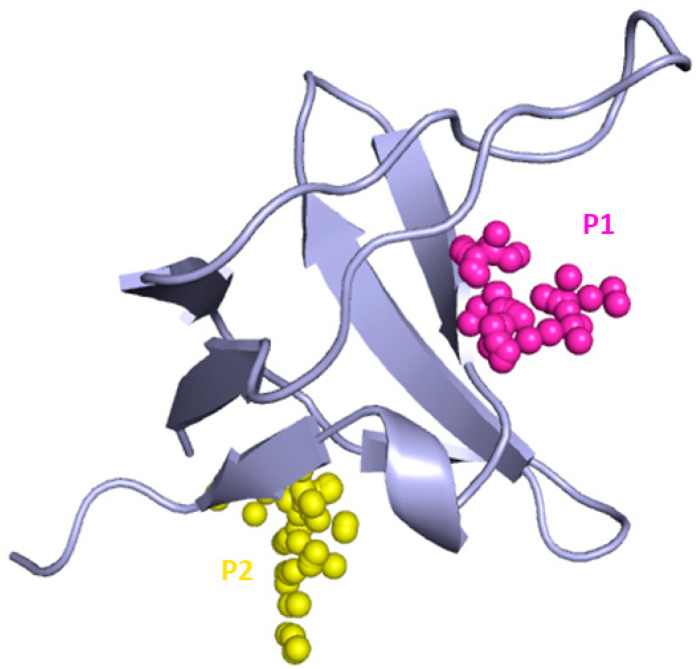
Binding pockets detected by F-Pocket. P1 is represented by magenta alpha spheres while P2 by yellow alpha spheres.

F-pocket analysis on the representative MD snapshots highlighted the presence of two putative druggable binding pockets named P1 and P2 (Figure 4).

**Figure 5:**
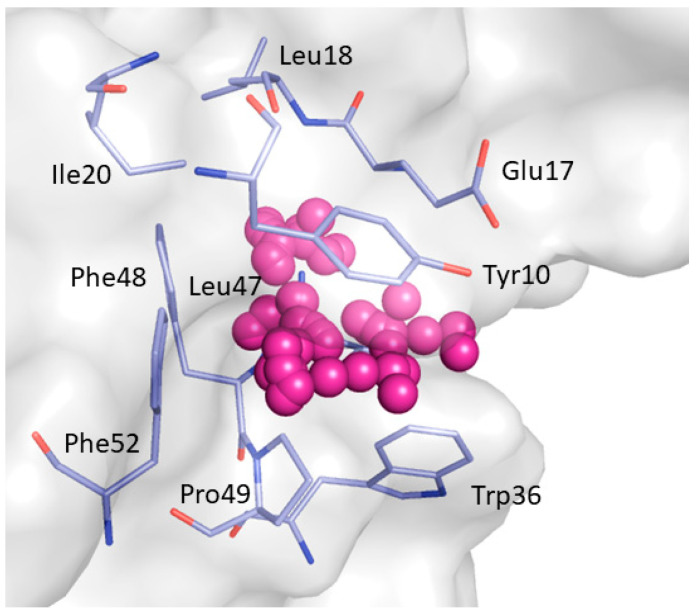
Close view of the binding pocket (P1) detected by F-pocket. Magenta alpha spheres represent P1. The residues lining P1 are shown as light blue sticks.

P1 was predicted for the representative frames of C8, C10, C11, C13, C14, and C19 with druggability score values ranging from 0.645 to 0.934. The pocket is situated at the interface of the SH3A domain of CIN85 involved in PPIs and gets formed between 170 and 230 ns of simulation time due to the movement of the RT loop. P1 is lined by Tyr10, Glu17, Leu18, Ile20, Trp36, Leu47, Phe48, Pro49, and Phe52 (Figure 5).

**Figure 6:**
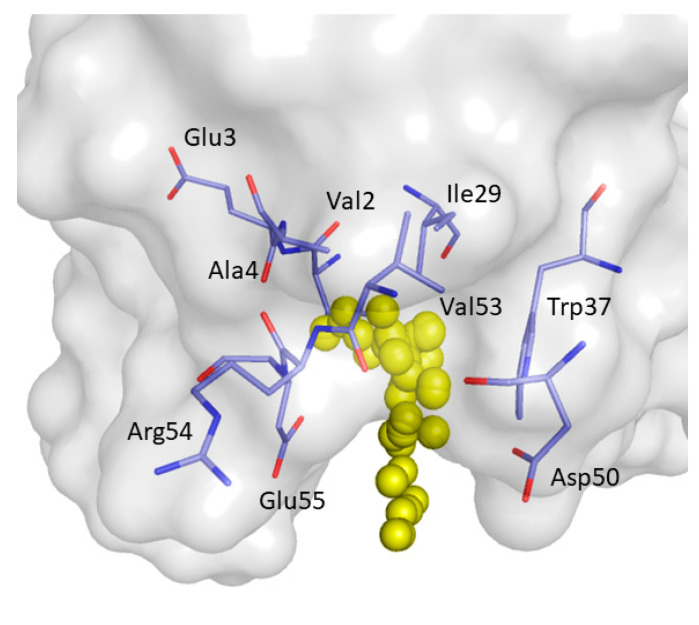
Close view of binding pocket P2 detected by F-pocket. Yellow alpha spheres represent P2. The residues forming P2 are shown as light blue sticks.

The druggable binding pocket P2 was identified in a region between strands β_1_, β_5_ and the 3_10_ helix in the representative snapshots of C12 and C16, for which druggability score values of respectively 0.6150 and 0.577 were estimated by F-pocket. P2 is lined by Val2, Glu3, Ala4, Ile29, Trp37, Asp50, Val53, Arg54, and Glu55 (Figure 6).

**Figure 7:**
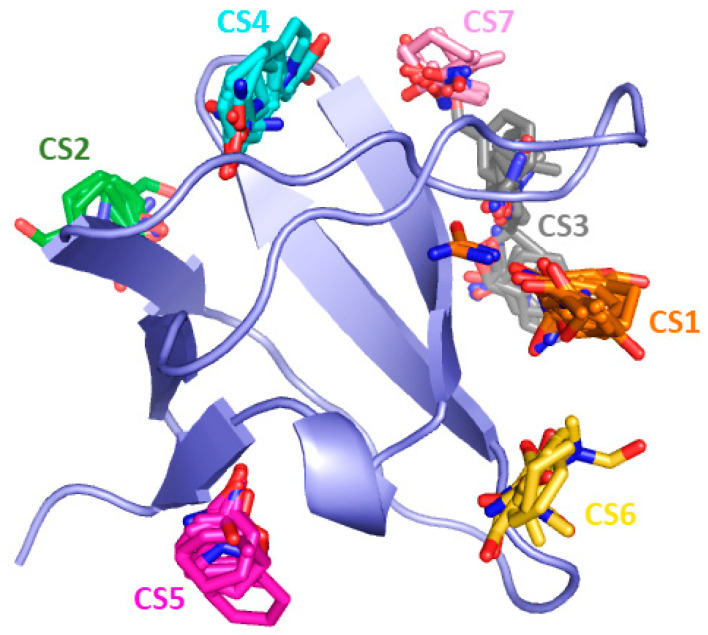
Consensus sites (CS) identified by FTMap. Probe clusters, which identified the hotspot regions, are represented as sticks. Probes belonging to the same CS are characterized by having the same color.

A binding site analysis on the representative conformers performed by means of FTMap webserver revealed the presence of seven druggable CSs which are shown in Figure 7.

**Figure 8:**
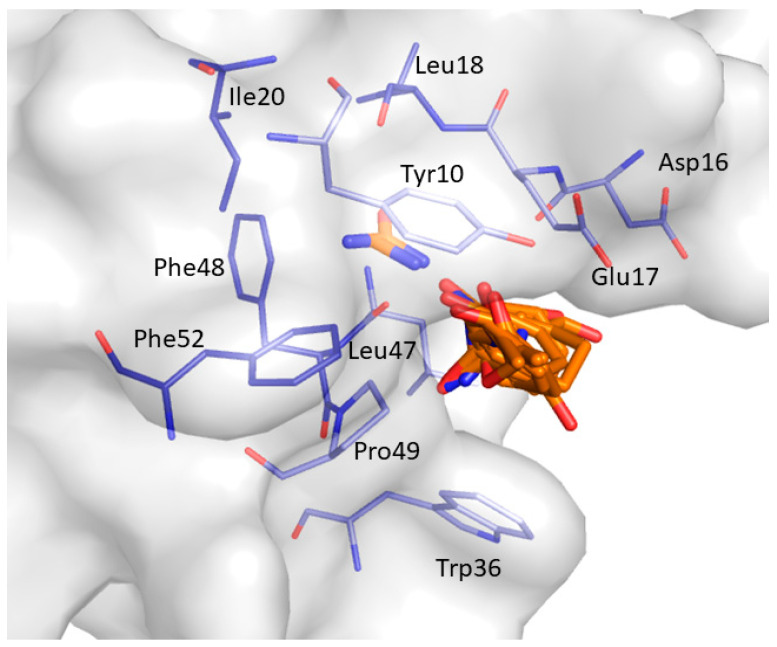
Most frequently occurring consensus site identified by FTMap. The representative probes docked and clustered by FTMap, that define the putative binding site, are displayed as orange sticks. The residues interacting with the probes are shown as light blue sticks.

The most frequently occurring binding site CS1 was retrieved for 20 clusters (C10-C13; C15-C22; C24; C26-32) and is shown in Figure 8. CS1 comprising the residues Tyr10, Asp16, Glu17, Leu18, Ile20, Trp36, Leu47, Phe48, Pro49, and Phe52 is located at the binding interface of the CIN85 SH3A domain. Interestingly, the location of CS1 matches quite well the location of pocket P1 predicted by F-pocket.

**Figure 9:**
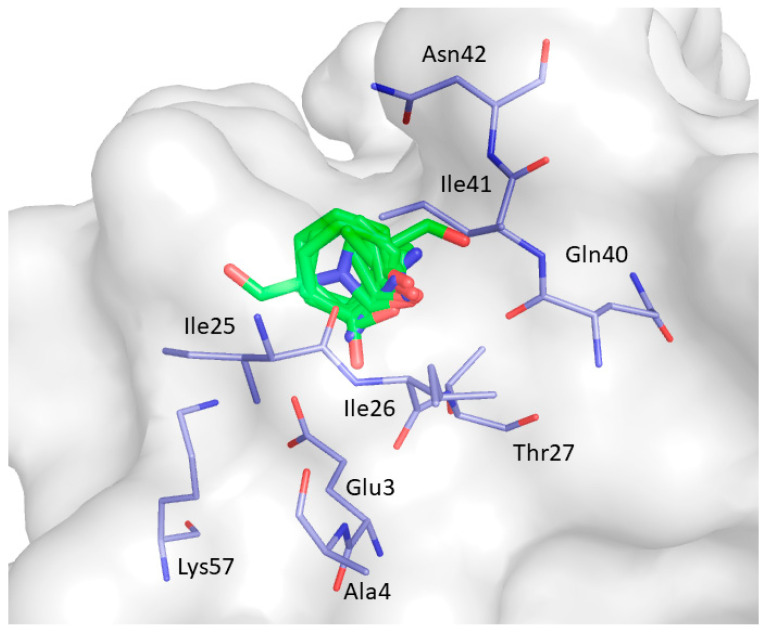
Consensus site CS2 predicted by FTMap. Clustered probes are depicted as green sticks.

CS2 was identified in 9 clusters (C7, C13, C22, C24, C26, C29-C32). It is located in the space between the distal loop, β_1_, and β_2_ strands. It comprises residues Glu3, Ala4, Ile25, Ile26, Thr27, Gln40, Ile41, Asn42, and Lys57respectively, and is displayed in Figure 9.

**Figure 10:**
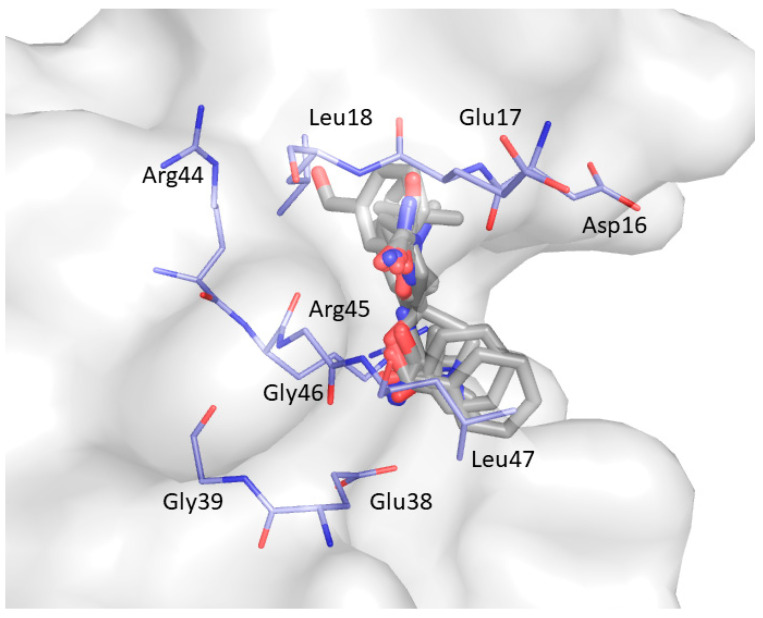
Consensus site CS3 predicted by FTMap. Clustered probes are represented as grey sticks.

CS3 was retrieved for 5 clusters (C10, C25-C26 and C29-C30). As shown in Figure 10, the probes are surrounded by Asp16, Glu17, Leu18, Glu38, Gly39, Arg44, Arg45, Gly46, and Leu47 located on the RT loop and the β_4_ strand.

**Figure 11:**
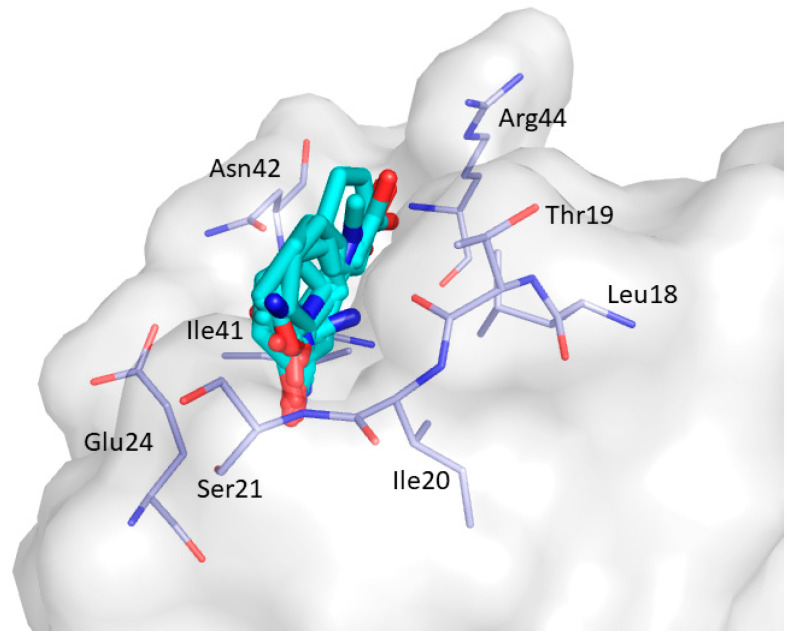
Consensus site CS4 predicted by FTMap. Clustered probes are shown as cyan sticks.

CS4 was found in the representative frames of 4 clusters (C1; C3; C12; C24). CS4 is located between the β_2_ strand, distal loop, and the RT loop and includes amino acid residues Leu18, Thr19, Ile20, Ser21, Glu24, Ile41, Asn42, and Arg44 (Figure 11).

**Figure 12:**
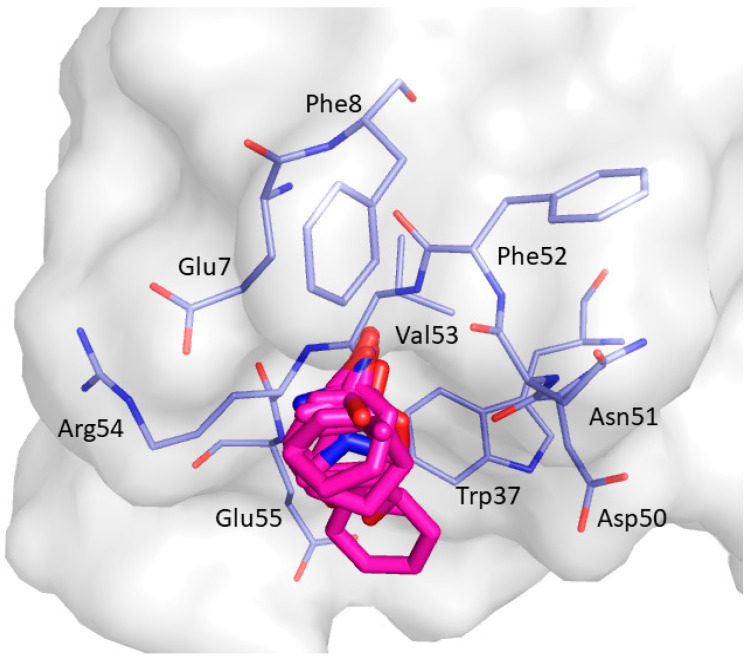
Consensus site CS5 predicted by FTMap. Clustered probes are shown as magenta sticks.

CS5, displayed in Figure 12, was detected in 4 clusters (C1; C6; C8; C12). It is located close to the β_2_ strand and comprises the amino acid residues Glu7, Phe8, Trp37, Asp50, Asn51, Phe52, Val53, Arg54, and Glu55.

**Figure 13:**
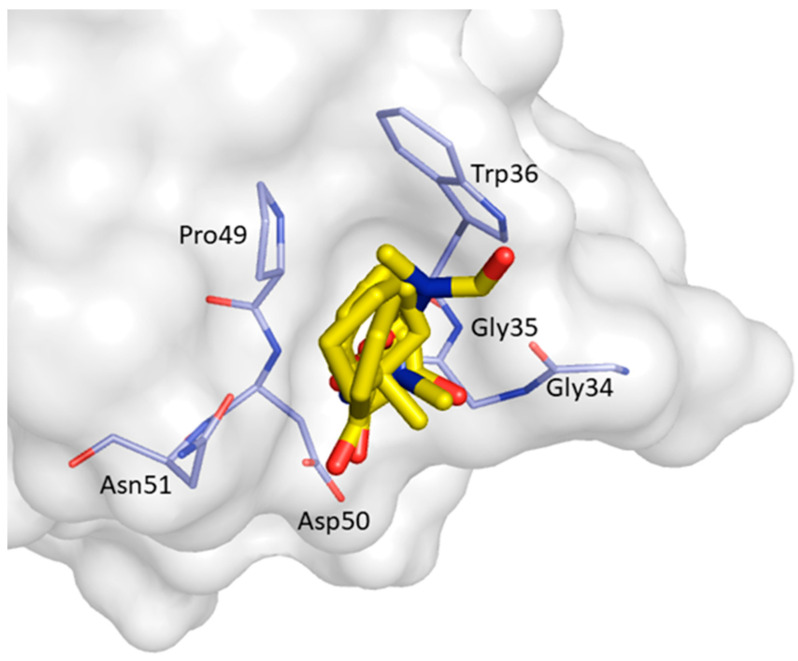
Consensus site CS6 predicted by FTMap. Clustered probes are shown as yellow sticks.

CS6 was predicted by FTMap in 2 clusters (C2; C9) and it located between the n-Src loop, 3_10_ helix, β_3_ and β_4_ strands. The clustered probes are surrounded by Gly34, Gly35, Trp36, Pro49, Asp50, and Asn51, and is shown in Figure 13.

**Figure 14:**
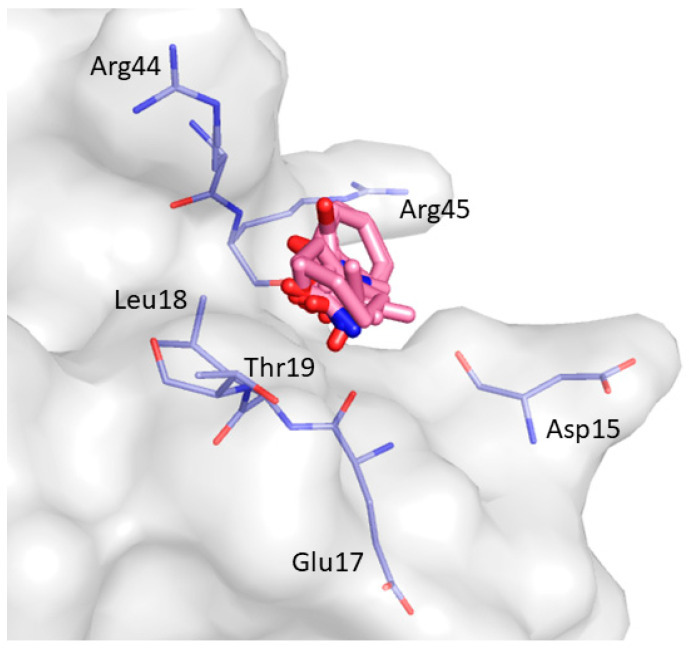
Consensus site CS7 predicted by FTMap. Clustered probes are shown as pink sticks.

Finally, the last binding site predicted by FTMap is CS7 which was identified in 2 clusters (C4; C7) between the RT loop and the β_4_ strand. This hotspot region includes Asp15, Glu17, Leu18, Thr19, Arg44, and Arg45 (Figure 14). 

For each of the reported binding sites, a list of the residues forming the pocket and the corresponding amino acids in the other SH3 domains of CIN85 is provided in Table S1 (see Supplementary Materials).

The visualization of clustered FTMap probes provides useful information about putative binding sites that can be exploited for the structure-based design of new ligands. Additionally, the program provides a list of possible hydrogen bonds and other non-bonded interactions established between the probes and the protein residues, giving further insights about the residues that should be targeted. In Table 1, we reported only the residues that account at least for 10% of all atomic-level probe-residue interactions over the 32 mapped protein conformations. Some of these residues (Tyr10 and Trp36) are conserved among SH3 domains and are involved in the recognition of Pro-rich peptides, so a targeting of these residues could result in the orthosteric inhibition of the PPI.

Accounting for protein flexibility allowed us to detect different putative binding sites predicted as being druggable by FTMap and F-pocket and demonstrated how protein motions promote the exposure of binding sites otherwise not detectable in a single static crystal structure. Particularly, FTMap identified 7 druggable binding sites. Among them, CS1 is located in the binding interface of the CIN85 SH3 domain and represents the most occurrent one. F-pocket analysis suggests that a druggable binding pocket P1 might be transiently opened between 170 and 230 ns of the simulation due to the movement of the RT loop in the same region of CS1; therefore, we hypothesize that this conformation could be stabilized by a small molecule able to occupy P1 pocket. The mapping by FTMap of the representative frames in which P1 was detected by F-Pocket showed, that various small molecular probes are able to bind to that pocket and finally resulted in a druggable consensus cluster. Molecules able to bind to this pocket thus might inhibit MUC1-CIN85 PPI with an orthosteric mechanism. The other binding sites identified by FTMap and F-pocket might accommodate ligands providing an allosteric inhibitory effect on MUC1-CIN85 PPI. 

## 3. Material and Methods

### 3.1. Molecular Dynamic Simulation and Frames Clustering

Explicit solvent molecular dynamics simulation was performed using the OpenMM implementation of CHARMM (Department of Chemistry and Chemical Biology, Cambridge, MA, USA) [26,30] starting from 3D coordinates of the human CIN85 SH3A domain extracted from the crystal structure of CIN85 in complex with the Cbl-b peptide (PDB code 2BZ8) [17]. The web-based graphical interface CHARMM-gui (http://www.charmm-gui.org/) [31] was used to set up the simulation and to generate the initial input files. The protein was solvated in a rectangular box using the TIP3P water model and neutralized with KCl setting a salt concentration of 0.15 M. After a 25 ps equilibration phase in NVT ensemble using a time step of 1 fs, the system was simulated in NPT ensemble for 400 ns with a timestep of 2 fs at 310 K by Langevin dynamics, while the pressure was kept around 1 atm by a Monte Carlo barostat. The coordinates were saved every 10 ps which resulted in 40,000 frames. Visualization and analysis of the trajectory were carried out by using the Visual Molecular Dynamics (VMD) software (NIH Center for Macromolecualr Modeling & Bioinformatics, Urbana-Champaign, IL, USA) [32]. The RMSD of the protein backbone was computed by means of the “RMSD Trajectory Tool” implemented in VMD. 

The obtained frames were clustered using TTClust (Institute for Integrative Biology of the Cell, Paris, France), a Python program for hierarchical clustering based on the RMSD of atomic coordinates. In particular, the program initially aligns the trajectory on the protein backbone. Subsequently, the RMSD between all atom pairs for each frame is computed and stored in a matrix which is then exploited to calculate a linkage matrix. Ward algorithm was chosen for the linkage matrix calculation. 

### 3.2. Binding Site Prediction

Binding site prediction was carried out for the crystal structure and for each representative protein conformation by employing F-pocket (Ressource Parisienne en BioInformatique Structurale, Paris, France) and the FTMap web server (https://ftmap.bu.edu). Only binding sites predicted as being druggable by the two methods were considered in this study. F-pocket and FTMap analyses were both performed using default parameter settings. 

## 4. Conclusions

Computer-aided drug discovery methods offer useful tools to study PPIs and to elucidate the characteristics of protein interfaces. This study was focused on MUC1-CIN85 PPI involved in the formation of metastasis in cancer cells. We report an in silico approach that was aimed at the identification of potential druggable binding sites on the SH3A CIN85 domain. Our idea was to provide useful hints for the structure-based design of novel ligands able to bind CIN85 and prevent its interaction with MUC1. To reach this goal, we incorporated protein flexibility by employing molecular dynamics simulations. Indeed, transient binding sites were formed through structural rearrangements that could not be observed in the static experimental structure. The resulting MD frames were clustered based on their RMSD and a representative conformation was selected from each cluster. Two binding site detection approaches were used. The first F-pocket was able to predict two putative binding pockets, while the second FTMap furnished a suggestion about seven druggable hotspot regions. Interestingly, both strategies revealed the presence of a druggable binding site on the CIN85 SH3A interface, that was not present in the crystal structure. Compounds able to bind this region might orthosterically inhibit MUC1-CIN85 PPI. Moreover, other potential binding sites were predicted as a valuable region to accommodate allosteric ligands. Overall, our work provided new structural insights about the CIN85 SH3 domain which may be useful for the identification of new leads through structure- and fragment-based methods. As an example, the identified binding sites might be exploited for docking studies with the resulting poses of the docked probes then serving as a starting point for the design of new ligands by fragment growing or fragment linking strategies.

## Figures and Tables

**Table 1:** Residues interacting with the mapping probes that account for at least the 10% of all the interactions over the 32 clusters.

Non-Bonded Interactions	H-Bonding Interactions
Phe8, Tyr10, Gln13, Asp16, Glu17, Leu18, Glu24, Ile25, Trp36, Trp37, Glu38, Asn42	Tyr10, Gln11, Gln13, Glu17 Thr19, Ser21, Ile25, Thr27, Asn42, Arg44, Arg45, Leu47, Asp50, Val53, Glu55

## Supplementary Materials

Supplementary materials can be found at https://www.mdpi.com/1422-0067/22/2/534/s1.

## Abbreviations

PPI	Protein-protein interaction
VNTR	Variable number of tandem repeats regions
SH3	Src homology 3
MD	Molecular dynamics
RMSD	Root mean square deviation
C	Cluster
CS	Consensus site
VMD	Visual molecular dynamics
