# Supplementary material for: In Silico Identification of Potential Druggable Binding Sites on CIN85 SH3 Domain
Source: Int J Mol Sci. 2021 Jan 7;22(2):534. doi: 10.3390/ijms22020534 (PMC7825788; doi:10.3390/ijms22020534)
Supplement: Supplementary file 1 [file ijms-22-00534-s001.pdf]

In silico identification of potential druggable binding sites on CIN85 SH3 domain

Serena Vittorio [1,*], Thomas Seidel [2], Arthur Garon [2], Rosaria Gitto [1], Thierry Langer [2], Laura De Luca [1,*]

[1] Department of Chemical, Biological, Pharmaceutical and Environmental Sciences, University of Messina, Viale Palatucci 13, I-98168, Messina, Italy; svittorio@unime.it (S.V.); rgitto@unime.it (R.G.); ldeluca@unime.it (L.D.L.)
[2] Department of Pharmaceutical Chemistry, University of Vienna, Althanstrasse 14, A-1090 Vienna, Austria; thomas.seidel@univie.ac.at (T.S.); arthur.garon@univie.ac.at (A.G.); thierry.langer@univie.ac.at (T.L.)

*Correspondence: svittorio@unime.it; Tel.: +39-090-676-6465 (S.V.); ldeluca@unime.it; Tel.: +39-090-676-6410 (L.D.L.)

Table of content:

TableS1 List of the residues forming the identified binding sites in CIN85 SH3A and corresponding amino acids in the other CIN85 SH3 domains obtained from 3D superimposition……………………………………..S2
FigureS1 Superimpositions between some of the NMR derived models contained in 2K9G and some of the MD derived conformers………………………………………………………………………….S3

Table S1 List of the residues forming the identified binding sites in CIN85 SH3A and corresponding amino acids in the other CIN85 SH3 domains obtained from 3D alignment.

**Binding site**	**CIN85 SH3A**	**CIN85 SH3B**	**CIN85 SH3C**
**P1**	Tyr10, Glu17, Leu18, Ile20, Trp36, Leu47, Phe48, Pro49, Phe52	Tyr109, Glu116, Leu117, Leu119, Trp135, Met146, Phe147, Pro148, Phe151	Tyr278, Glu285, Leu286, Ile288, Trp306, Val317, Phe318, Pro319, Phe322
**P2**	Val2, Glu3, Ala4, Ile29, Trp37, Asp50, Val53, Arg54, Glu55	Arg101, Arg102, Cys103, Val127, Trp136, Ser149, Ile52, Lys153, Glu154	Asp270, Tyr271, Cys272, Leu296, Trp307, Asp320, Val323, Lys324, Leu325
**CS1**	Tyr10, Asp16, Glu17, Leu18, Ile20, Trp36, Leu47, Phe48, Pro49, Phe52	Tyr109, Asp115, Glu116, Leu117, Leu119, Trp135, Met146, Phe147, Pro148, Phe151	Tyr278, Asp284, Glu285, Leu286, Ile288, Trp306, Val317, Phe318, Pro319, Phe322
**CS2**	Glu3, Ala4, Ile25, Ile26, Thr27, Gln40, Ile41, Asn42, Lys57	Arg102, Cys103, Ile124, Ile125, Glu126, Val139, Leu140, Asn141, Ser156	Tyr271, Cys272, Ile293, Val294, Thr295, Glu310, Leu311, Asn312, Pro327
**CS3**	Asp16, Glu17, Leu18, Glu38, Gly39, Arg44, Arg45, Gly46, Leu47	Asp115, Glu116, Leu117, Glu137, Gly138, Lys143, Thr144, Gly145, Met146	Asp284, Glu285, Leu286, Glu308, Gly309, Arg314, Arg315, Gly316, Val317
**CS4**	Leu18, Thr19, Ile20, Ser21, Glu24, Ile41, Asn42, Arg44	Leu117, Glu118, Leu119, Lys120, Asp123, Leu140, Asn141, Lys143	Leu286, Thr287, Ile288, Lys289, Asp292, Leu311, Asn312, Arg314
**CS5**	Glu7, Phe8, Trp37, Asp50, Asn51, Phe52, Val53, Arg54, Glu55	Ala106, Phe107, Trp136, Ser149, Asn150, Phe151, Ile152, Lys153, Glu154	Ile275, Phe276, Trp307, Asp320, Asn321, Phe322, Val323, Lys324, Leu325
**CS6**	Gly34, Gly35, Trp36, Pro49, Asp50, Asn51	Glu133, Gly134, Trp135, Pro148, Ser149, Asn150	Val304, Gly305, Trp306, Pro319, Asp320, Asn321
**CS7**	Asp15, Glu17, Leu18, Thr19, Arg44, Arg45	Asp114, Glu116, Leu117, Glu118, Lys143, Thr144	Asp283, Glu285, Leu286, Thr287, Arg314, Arg315

FigureS1 Superimpositions between some of the NMR derived models contained in 2K9G and some of the MD derived conformers.

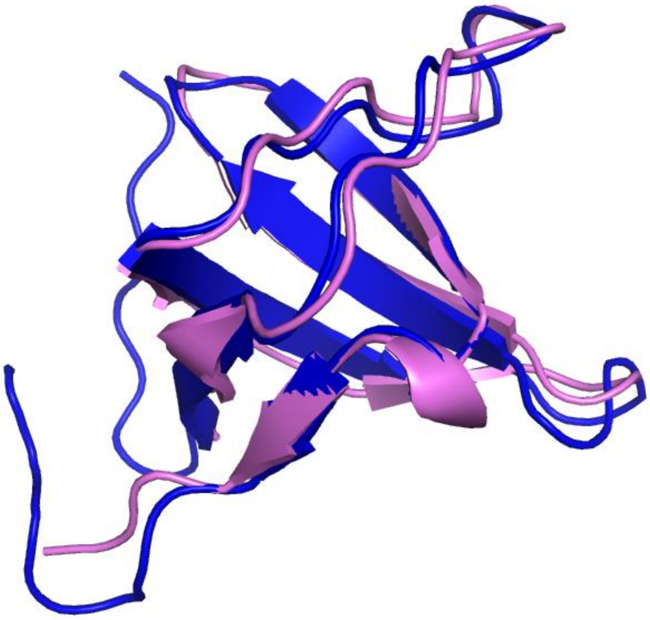

A) Model 1 of 2K9G (blue) superimposed to the representative frame of C2 (violet) (RMSD= 1.090 Å)

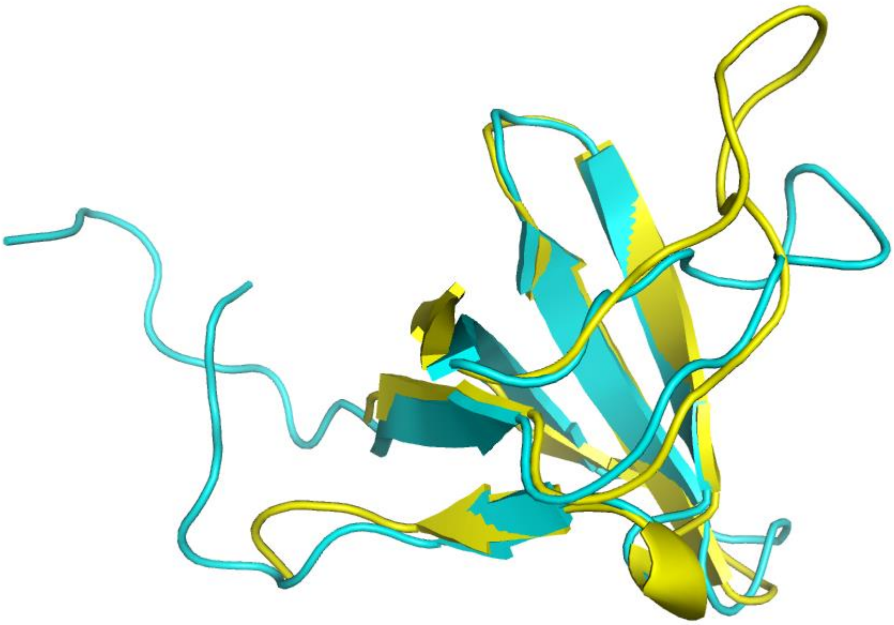

B) Model 2 of 2K9G (cyan) overlapped to the representative frame of C27 (yellow) (RMSD= 1.507 Å)

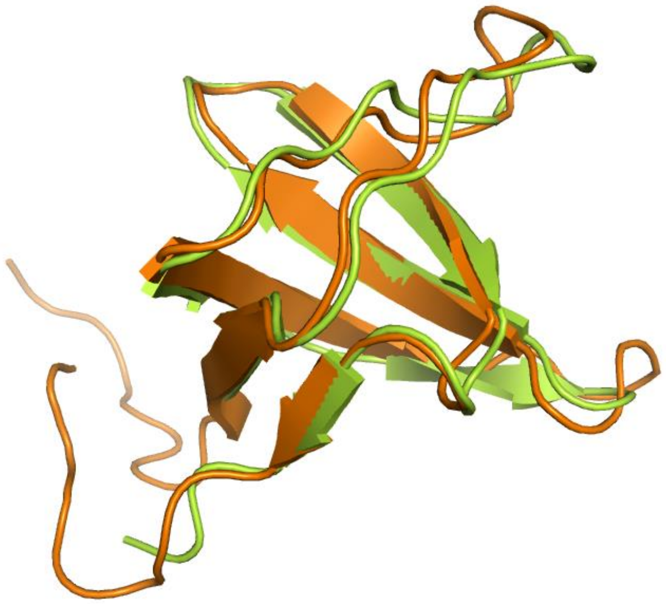

C) Model 3 of 2K9G (green) superimposed to the representative frame of C1 (green) (RMSD= 1.163 Å)

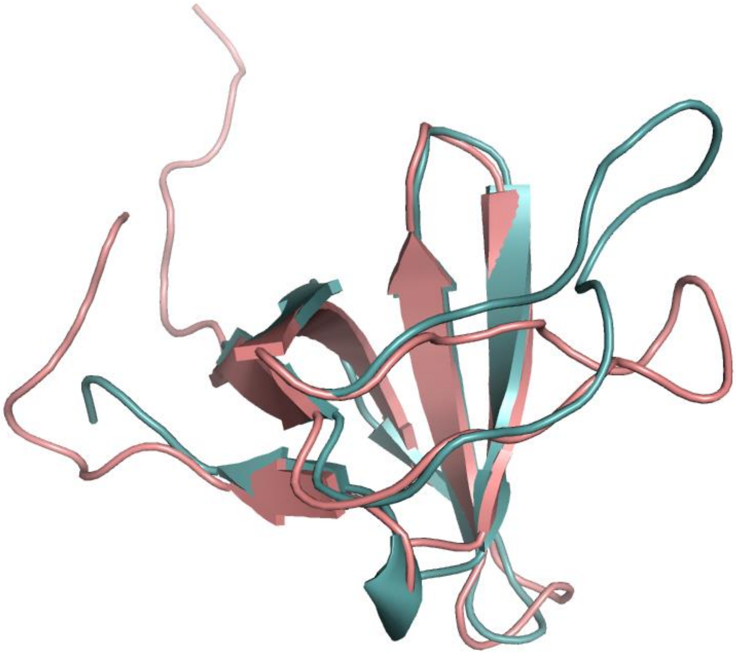

D) Model 5 of 2K9G (pink) superimposed to the representative frame of C26 (palecyan). (RMSD= 1.612Å)

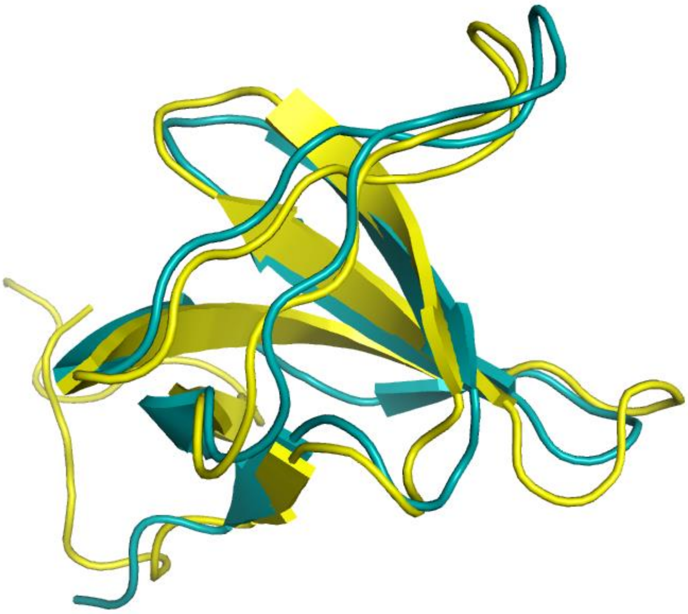

E) Model10 of 2K9G (giallo) superimposed to the representative frame of C10 (deepteal) (RMSD= 1.931 Å).

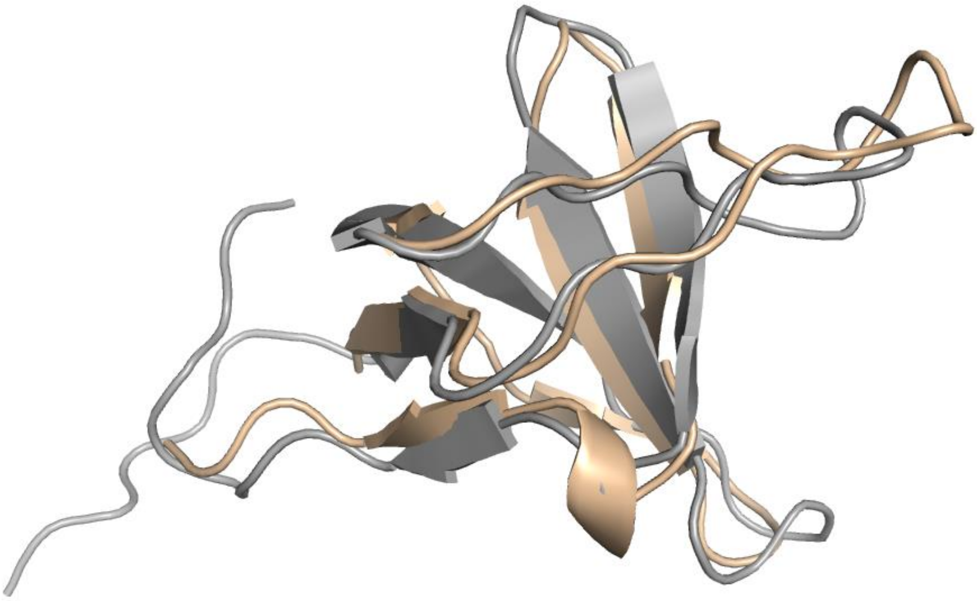

F) Model14 of 2K9G (grey) superimposed to the representative frame of C12 (wheat). (RMSD= 2.498Å)

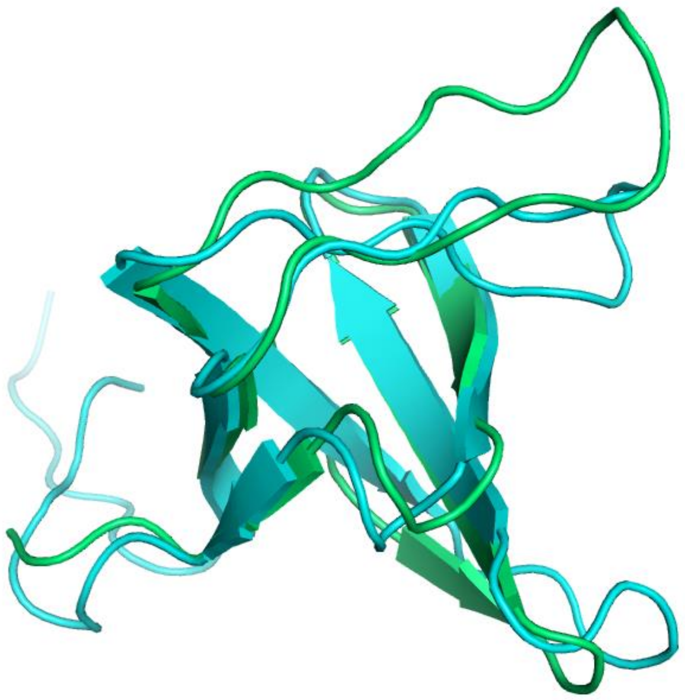

G) Model16 of 2K9G (cyan) superimposed to the representative frame of C17 (green) (RMSD = 1.777 Å)

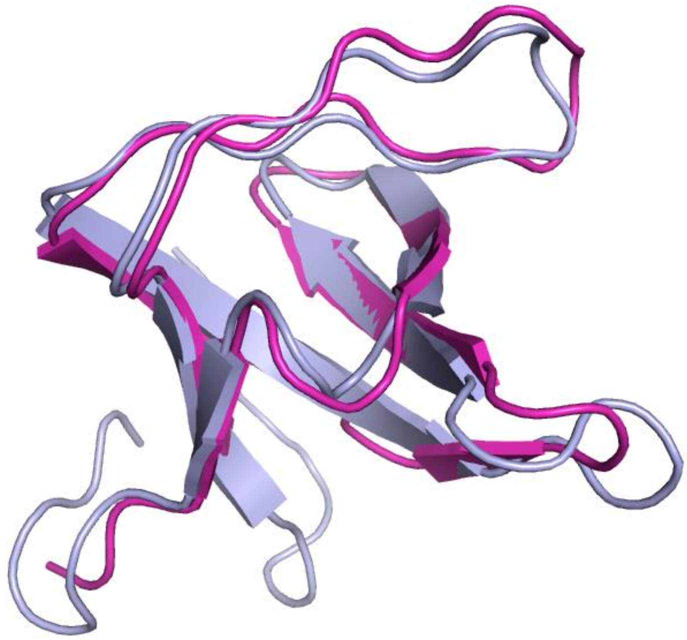

H) Model20 of 2K9G (lightblue) superimposed to the representative frame of C6 (magenta) (RMSD = 0.914).
